# Cancer and the Search for Selective Biochemical Inhibitors

**DOI:** 10.1038/sj.bjc.6604993

**Published:** 2009-03-31

**Authors:** D L Selwood

**Affiliations:** 1 The Wolfson Institute for Biomedical Research, University College London, London, UK

I think I should start this review with a quote from this book from the section ‘the ponderables of biochemistry’.

P15

‘Beyond this, however we are adrift (discussion of electrochemistry of the body and biochemical processes), as the mysteries that are the spark of life remain untouched. In other words, what distinguishes a live cell form a dead one, a live virus from a killed virus? Or more completely, what in the ultimate analysis distinguishes a live human being from a dead one? Can there be, or cannot there be, an inherent bio-physico-magneto-electro-chemical explanation at the most fundamental level? Or are we speaking of another domain, entirely, for example metaphysics, literally beyond or apart from science?’

There is much much more of this for a full 420 pages, indeed a shorter book would have been very welcome. Given that this is a book about alternative therapies for cancer, it is perhaps unlikely to appeal to the career scientist. It is common for example to find bizzare theories repeated, such as ‘pleomorphism’ p68, which supposes that harmless bacteria can become disease-producing viruses. Multiple sources are provided for each theory, but one must assume that these are not peer-reviewed journals. The merits (or lack of them) of megadoses of vitamin C are given another airing here. In fact, the book concludes with a recommendation of vitamin C as a cheap therapy for cancer.

With such a long book, I have inevitably to be selective while reviewing. If we look at a section entitled ‘The Navajo and Cancer’ (p351), the author makes a connection between viruses in food (in this case in poultry) being a cause of cancer, and the taboo on raising and eating poultry in the Navajo as a factor in the low incidence of cancer. He also has some interesting observations. He concludes the second paragraph with

‘Inasmuch as a rigorous system of classification has not been developed for viruses, they are categorized by size and shape and include picornaviruses, reoviruses, adenoviruses, caliciviruses, astroviruses, etc. An unknown factor is whether or not they may transmute to retroviruses, considered a prime factor in cancer causation..’

Well size and shape might once have defined virus classification, but genome sequence analysis long ago replaced such methods. As for viruses with genetic material of one type transmuting into retroviruses?

As for the proposal that the low incidence of cancer is related to poultry consumption, I searched pubmed (the US National library of medicine database that contains 18 million citations for biomedical articles dating back to 1948). There is indeed an article entitled ‘Cancer immunity in the Navajo’ by CG Salsbury dating from 1956 and other articles suggest that cancer incidence is lower among the Navajo than in the general population (see a commentary by D Espey *et al* (2007): National report to the nation regarding cancer, 1975–2004, featuring cancer in american indians and alaska natives. *Cancer*, 2007, 110, 2119–2152). However, multiple searches for articles or studies linking cancer with chicken consumption in the Navajo revealed nothing. Indeed although some epidemiological studies have linked meat consumption with cancers in the general population conflicting findings have been reported. So there seems to be no back up in terms of hard science for this link of low cancer in the Navajo to chicken consumption.

The worst thing about this is that there is a serious and legitmate discussion to have about the number of cancers caused by viruses and bacteria. Probably 15–20% of human cancers are caused by viruses. Thus, we can think not only of retroviruses, but herpes viruses, such as human herpes virus 8, which is the causative agent of Kaposi's sarcoma, or Hepatitis B and C virus – liver cancers, papilloma virus – cervical cancers. Of course, the bacterium *Helicobacter pylori* was identified as the causative agent in some stomach cancers. There is plenty of rock solid evidence about viruses, bacteria and cancer without the need to bring up a theory that has little information to support it.

With this title one might suppose that the book would lay out biochemical pathways, at least in diagrammatic form, to illustrate how inhibitors act. Sadly, this is not the case. There are only two pathways given at all where surely appropriate use of diagrams and colour would have greatly simplified the discussions at many points – causes of cancer for example. The book is also not strong on detailed biochemistry or chemistry; the table on vitamins and hormones as enzyme inhibitors is a case in point. The various inhibitors are simply listed, but without the sources in the table and also critically without the potency observed. A millimolar strength inhibiton is unlikely to have any therapeutic significance, a nanomolar one might. The table lists the molecular formula for each inhibitor, data of no conceivable value to the argument. There are also outright errors, for example p418 line 11, ‘ the presence of cancer cells in blood serum may be indicated by the Ames test’ really? I thought the Ames test was to determine the potential of chemicals to cause genetic abnormalities and is conducted on bacteria.

That said, the section in the book on surveying anticancer plant substances, p233, does provide a survey of the literature, which might prove useful for some investigators. There is a review of Judah Folkman's work on angiogenesis. If the author kept to solid facts, there might be a reasonable text in here. It is perfectly possible to write about plant products and their action against cancers, particularly when one considers how some have been modified to improve their properties for application in the clinic. However, even when there is a good story to tell, the author does not tell it: taxol (paclitaxel), for example (page 212, last paragraph), is discussed briefly as follows:

‘Also there is Taxol, derived from the bark of the Pacific yew, an evergreen tree or shrub, notably of the Pacific Northwest. (Yew wood was once favored by the Indians for bows and other purposes.) If successful, its relative scarcity *versus* demand indicates that chemistry will have to come to the rescue, especially the speciality known as organic chemistry’

Taxol was approved for ovarian cancer in 1992, and is now used to treat ovarian, breast and non-small cell lung cancer. Taxol is now isolated from a specific *Taxus* cell line cultured to produce the drug (by the Phyton Biotech company, Ahrensburg, Germany). This replaced the earlier semisynthetic process. Taxol is a sucessful antitumour drug, and there is no issue with supply. So this paragraph is woefully out of date.

I struggle to think who would read this book; patients would be best advised to contact their doctor/physician and look for information on the websites of the major cancer organizations such as Cancer Research UK. Perhaps chemists interested in plant products might scan the lists of sources, but that is all.

